# Age-Specific Association Between Urinary Phthalate Metabolites and Diabetes Mellitus: Findings from the Korean National Environmental Health Survey Cycle 4 (2018–2020)

**DOI:** 10.3390/healthcare14050655

**Published:** 2026-03-05

**Authors:** Jung-Eun Lee, Gyu Tae Lee, Han-A Cho

**Affiliations:** 1 Department of Counseling Psychology, Shinhan University, Uijeongbu 11644, Gyeonggi-do, Republic of Korea; jelee@shinhan.ac.kr; 2 Department of Big Data Management, Shinhan University, Uijeongbu 11644, Gyeonggi-do, Republic of Korea; lgstar728@naver.com; 3 Department of Dental Hygiene, Shinhan University, Uijeongbu 11644, Gyeonggi-do, Republic of Korea

**Keywords:** phthalates, urinary metabolites, diabetes mellitus, endocrine-disrupting chemicals, KoNEHS, complex survey design, age-stratified analysis, environmental health equity, elderly living alone

## Abstract

**Highlights:**

**What are the main findings?**

- Urinary phthalate metabolites showed distinct association with diabetes mellitus across age groups, indicating substantial age-related heterogeneity.
- MnBP and MEP emerged as key metabolites, with a strong association in young and older adults, whereas their effects in middle-aged adults were more moderate.

**What are the implications of the main findings?**

- Age should be considered an effect modifier, rather than merely a covariate, in environmental diabetes research.
- Age-tailored exposure prevention and surveillance strategies may enhance the effectiveness of diabetes prevention efforts.

**Abstract:**

**Background/Objectives**: Phthalates are encountered in everyday consumer and indoor environments, and their metabolites are commonly detected in urine. Although phthalate exposure has been linked to diabetes mellitus (DM), associations may vary by life stage. Therefore, we evaluated age-specific association between urinary phthalate metabolites and DM using nationally representative Korean data. **Methods**: We conducted a cross-sectional analysis of the Korean National Environmental Health Survey Cycle 4 (2018–2020). Adults aged ≥19 years with complete data were included. Eight urinary metabolites were evaluated. Metabolites were log-transformed, and those showing interaction were analyzed by tertiles. Complex survey-weighted logistic regression estimated odds ratios (95% confidence intervals) for DM, adjusting for demographic, socioeconomic, and health behavior factors. Analyses were stratified by age group. **Results**: Geometric mean (GM) concentrations among participants with DM varied significantly by age groups for several metabolites. Interaction analyses identified statistically significant effects for selected phthalate metabolites, including MnBP, MCPP, and MEP. In the age-stratified adjusted models, MnBP and MCPP were more strongly associated with DM in young adults, whereas the pattern for MEP appeared more evident in older adults, suggesting potential life-course differences in metabolic vulnerability. **Conclusions**: Associations between urinary phthalate metabolites and DM vary substantially by age, indicating life-course differences in exposure pathways and metabolic vulnerability. Age-specific prevention and surveillance strategies may improve environmental health interventions for DM.

## 1. Introduction

One of the most pressing public health challenges in the twenty-first century is the rapid and sustained increase in the incidence of diabetes mellitus (DM). Recent reports from the International Diabetes Federation and World Health Organization (WHO) have indicated that the global prevalence of diabetes among adults is 14% as of 2022. Diabetes-related mortality has continued to increase since 2000, in contrast to the declining probability of premature death from other major noncommunicable diseases, such as cardiovascular diseases and cancer [1]. Of particular concern is the growing incidence of youth-/early-onset type 2 diabetes (T2D) among children, adolescents, and young adults. Early-onset T2D is associated with longer disease duration and increased likelihood of early complications, thereby increasing its priority as a critical public health issue [2]. These epidemiological shifts are difficult to explain solely by conventional risk factors, such as obesity, dietary patterns, and physical inactivity. Consequently, increasing attention has been directed toward endocrine-disrupting chemicals (EDCs), often referred to as environmental “obesogens,” as potential hidden contributors to metabolic homeostasis disruption and the development of metabolic disorders [3,4].

Phthalates have been extensively used as plasticizers and solvents to enhance the flexibility of plastic materials and are ubiquitous in modern environments. Human exposure occurs through multiple everyday pathways, including personal care products, cosmetics, food packaging materials, household goods, and indoor building materials [5,6,7]. Because of their widespread use and continuous exposure, phthalates are among the most frequently detected environmental chemicals in human biomonitoring studies. Urinary metabolites, such as monoethyl phthalate (MEP), mono-n-butyl phthalate (MnBP), and mono(3-carboxypropyl) phthalate (MCPP), are commonly used as biomarkers of phthalate exposure and are widely used in epidemiological research [8,9].

Epidemiological studies have repeatedly suggested a potential association between phthalate exposure and T2D or abnormalities in glucose metabolism. Analyses based on the National Health and Nutrition Examination Survey (NHANES) have reported a positive association between selected urinary phthalate metabolites and both diabetes prevalence and markers of insulin resistance [10]. In addition, recent review articles have summarized the biological plausibility linking phthalate exposure to metabolic disorders, including diabetes [4]. Mechanistically, phthalates interact with peroxisome proliferator-activated receptors, thereby promoting adipocyte differentiation, disrupting insulin signaling pathways, and inducing oxidative stress in pancreatic β-cells, which may collectively increase the risk of diabetes development [11,12].

Certain phthalate monoester metabolites, including mono(2-ethylhexyl) phthalate (MEHP) and monobenzyl phthalate (MBzP), may plausibly influence metabolic homeostasis via modulation of nuclear receptor signaling. In particular, the peroxisome proliferator-activated receptor (PPAR) axis—comprising PPARα and PPARγ—constitutes a central regulatory hub linking lipid handling, adipocyte differentiation (adipogenesis), and insulin sensitivity. Toxicological evidence indicates that phthalate monoesters can engage and activate these receptors, supporting an endocrine-disrupting mechanism relevant to dysmetabolism [13]. In addition, experimental findings suggest that phthalate exposure can exacerbate T2D-related phenotypes by perturbing the canonical insulin signaling cascade, notably the PI3K/AKT pathway, and by downregulating pancreatic GLUT2 (SLC2A2), thereby impairing glucose sensing and insulin secretory dynamics [14]. Collectively, these mechanistic strands converge on a coherent pathway framework—endocrine disruption with altered lipid metabolism, followed by insulin signaling impairment and heightened β-cell vulnerability—providing biologically grounded plausibility for epidemiologic associations between urinary phthalate metabolites and diabetes.

A major limitation of the existing epidemiologic literature is that age has frequently been treated merely as an adjustment covariate or, alternatively, adult populations have been analyzed as a single homogeneous stratum [4], thereby limiting rigorous evaluation of age as an effect modifier of the exposure–disease relationship. From a life-course perspective, aging is accompanied by coordinated shifts in body composition, endocrine milieu, renal clearance, and broader metabolic capacity, as well as cumulative inflammatory and oxidative stress burden. These age-related physiologic transitions can simultaneously alter both the internal dose (i.e., toxicokinetic handling and biologically effective exposure) and metabolic susceptibility (i.e., vulnerability of glucose-regulatory systems to perturbation), providing a strong conceptual rationale for explicitly testing age-stratified associations and interaction effects in phthalate–diabetes research [15,16,17].

DM may exhibit etiologic heterogeneity by age at onset [18], and therefore, the magnitude and even the direction of the phthalate–DM association may not be uniform across population subgroups [4,19]. In parallel, dominant sources and exposure profiles of phthalates are likely to differ by age group [6,8,20]. Taken together, these considerations underscore the need for nationally representative biomonitoring studies that evaluate phthalate–DM associations using age-stratified analyses.

Accumulating evidence further suggests that youth-onset T2D follows a more aggressive clinical course than adult-onset disease, characterized by more rapid β-cell functional decline, earlier treatment failure, and earlier accumulation of diabetes-related complications [21]. In addition, progression from prediabetes to T2D diabetes may occur over a relatively short period in adolescents, raising the possibility that the duration and phenotypic expression of the prediabetic stage are not directly comparable to those observed in adults [22]. In the context of the increasing incidence of early-onset T2D [2], empirically investigating the contribution of environmental factors among younger populations has important public health implications.

A review of prior studies indicates that existing research has largely focused on sex-specific analyses [20,23], gestational diabetes among pregnant women [4,24], or associations between phthalates and individual disease outcomes [10,25,26,27]. However, most of these studies analyzed the general adult population as a single group or considered age solely as an adjustment variable, thereby neglecting life-course-specific exposure pathways and biological susceptibility.

Furthermore, population-representative evidence has been reported primarily from a limited number of countries, such as the United States [10,27] or the Republic of Korea [20,23]. Studies that use nationally representative data to examine age-stratified associations between phthalate exposure and diabetes while simultaneously incorporating socioeconomic factors into their interpretation remain scarce. Therefore, the present study aimed to investigate the association between phthalate exposure and diabetes across different age groups using data from the 4th Korean National Environmental Health Survey (KoNEHS) (2018–2020), the most recent nationally representative dataset available. Based on these findings, this study seeks to provide evidence-based implications for public health policies and environmental health interventions. This approach addresses a key limitation of prior analyses that implicitly treated adult populations as homogeneous and provides an evidence base for developing life-course-tailored exposure prevention and risk surveillance strategies.

## 2. Methods

### 2.1. Study Design and Data Source

This study used a cross-sectional design using raw data from the KoNEHS Cycle 4. The KoNEHS is a nationwide, government-led survey administered by the Ministry of Environment and the National Institute of Environmental Research (NIER) to assess population-level exposure to environmentally hazardous substances and the associated health outcomes among Korean adults. The survey was approved by the NIER Institutional Review Board (approval no. NIER-2018-BR-003-02), and the dataset was provided under restricted access in accordance with the approved research purpose and data-use policies.

The KoNEHS is one of Korea’s nationally approved statistics and follows a protocol comparable to that of the NHANES. In accordance with the KoNEHS sampling framework, a stratified multistage cluster sampling design and complex survey procedures were used. To obtain nationally representative estimates, all analyses incorporated survey design variables, including strata, primary sampling units (clusters), and sampling weights.

### 2.2. Study Population

The analytic sample comprised adults aged ≥19 years who participated in the KoNEHS Cycle 4 (2018–2020) adult survey. Among eligible participants, 4239 individuals with complete information on key study variables, including urinary phthalate metabolite concentrations, DM status, and general characteristics/covariates, were included in the final analysis. For biomonitoring, urine specimens (approximately 50–75 mL) were collected from participants who provided written consent for research involving human biological materials. Age was categorized into three groups (19–39, 40–64, and ≥65 years), and all analyses were stratified by age group to examine age-specific associations.

### 2.3. Variable Definitions

This study used the urinary concentrations of phthalate metabolites as exposure indicators to examine the association between phthalate exposure and diabetes. Eight urinary phthalate metabolites were included: mono(2-ethyl-5-hydroxyhexyl) phthalate (MEHHP), mono(2-ethyl-5-oxohexyl) phthalate (MEOHP), MnBP, mono(2-ethyl-5-carboxypentyl) phthalate (MECPP), monobenzyl phthalate (MBzP), MCPP, MEP, and monomethyl phthalate (MMP). Values below the method detection limit (MDL) were imputed using MDL/√2 [23]. All metabolite concentrations were log-transformed prior to analysis to reduce right-skewness and improve the model fit. For logistic regression analyses, each metabolite was additionally categorized into tertiles representing low (T1), medium (T2), and high exposure (T3), following the approach used in a previous study [27].

DM status was the dependent variable and was ascertained based on HbA1c values obtained from the KoNEHS data. We categorized participants into two groups: the DM group (HbA1c ≥ 6.5%) and the non-DM group (HbA1c < 6.5%).

The independent variables included sex, socioeconomic indicators, social vulnerability, and health-related behaviors. Sex was categorized as male or female. Educational level and household income level were included to evaluate the potential contribution of socioeconomic factors to environmental inequality. Educational level was categorized as middle school or less, high school, or college and above. Household income level was defined using average monthly household income and categorized into four groups: <3.0 million KRW, 3.0–5.0 million KRW, 5.0–7.0 million KRW, and ≥7.0 million KRW. As an indicator of social vulnerability, marital status was dichotomized according to the presence of a spouse or a cohabiting partner (yes or no).

Lifestyle covariates, known to be major confounders of metabolic disorders, were also included. Current smoking was classified into current smoker or nonsmoker. High-risk drinking was defined as follows: for men, drinking on ≥4 days per week and consuming ≥5 drinks per occasion, and for women, drinking on ≥3 days per week and consuming ≥5 drinks per occasion. The participants who met these criteria were classified as high-risk drinkers. Because the KoNEHS 4th cycle (2018–2020) did not provide sufficiently detailed information on dietary patterns, biomarkers capturing individual oxidative stress, or precise physical activity measures, these factors could not be fully incorporated as covariates. As a result, residual confounding cannot be ruled out, which may limit more accurate source attribution and the interpretation of potential iatrogenic pathways.

### 2.4. Statistical Analysis

All statistical analyses were performed using R software (version 4.3.1; R Foundation for Statistical Computing, Vienna, Austria). To account for the complex survey design, the survey package was used. Prior to the analysis, a complex sampling design object was created by specifying the survey design variables, including strata, clusters (primary sampling units), and sampling weights.

Descriptive statistics were used to summarize the distributions and characteristics of the study variables. Cross-tabulations were performed to examine associations between diabetes status and the independent variables. In addition, the mean urinary concentrations of phthalate metabolites were calculated for individuals with diabetes in each age group.

To evaluate whether the association between phthalate exposure and the prevalence of DM differed across life stages, interaction tests were performed by including interaction terms between urinary phthalate metabolites (categorized by tertiles) and age groups in the complex survey-weighted logistic regression models. Subsequently, for phthalate metabolites showing statistically significant interaction effects, complex survey–weighted logistic regression models were fitted to estimate the odds of developing diabetes according to metabolite exposure tertiles. To account for multiple comparisons and testing, the Bonferroni correction was applied across the eight types of urinary phthalate metabolites evaluated in this study, defining statistical significance at a *p* < 0.00625 (0.05 divided by eight) for all associations with DM. Statistical significance was determined using a two-sided alpha level of 0.05.

## 3. Results

### 3.1. General Characteristics of the Study Population

A total of 4239 participants were included in the final sample. Of these, 980 (35.3%) were aged 19–39 years, 2247 (46.3%) 40–64 years, and 1012 (18.4%) ≥65 years (Table 1). The overall sex distribution was nearly balanced (49.8%, male; 50.2%, female); however, the proportion of female increased with age (19–39 years, 47.4%; 40–64 years, 50.1%; ≥65 years, 55.7%). Overall, the most common educational attainment was ≥college (51.6%), although marked differences were observed across age groups. In the 19–39-year group, 81.9% had ≥college education, whereas in the ≥65-year group, ≤middle school education accounted for 64.3% of the participants. Regarding household income, 40.6% of the total sample reported a monthly household income of <3.0 million KRW; this proportion was particularly high among those aged ≥65 years (81.0%), indicating comparatively lower income levels in older adults.

With respect to marital status, 66.9% of participants reported having a spouse or cohabiting partner, with the highest prevalence observed in the 40–64-year age group (87.2%). In contrast, the 19–39-year age group was predominantly characterized by the absence of a spouse/cohabiting partner (61.3%). The overall prevalence of current smoking was 18.7% and was highest among participants aged 40–64 years (21.1%). The prevalence of high-risk drinking was 10.1% in the total sample, peaking in the 40–64-year age group (12.9%) and reaching the lowest level among those aged ≥65 years (4.2%). Overall, 9.1% of the participants were classified as having DM, and diabetes prevalence substantially increased with age (19–39 years, 2.1%; 40–64 years, 10.9%; ≥65 years, 18.6%).

### 3.2. Differences in Participant Characteristics According to Diabetes Status

Among the total study population, 325 (9.1%) participants were classified as the DM group, and diabetes prevalence increased with age (19–39 years, 1.7%; 40–64 years, 10.8%; ≥65 years, 16.5%) (Table 2). Diabetes prevalence differed by sex; males exhibited a higher prevalence (11.5%) than females (6.7%). This sex disparity was most pronounced in the 40–64-year age group (male: 15.3% vs. female: 6.6%, *p* < 0.001). A clear socioeconomic gradient was observed. The prevalence of diabetes increased with decreasing educational attainment, ranging from 3.8% among those with ≥college education to 17.3% among those with ≤middle school education (*p* < 0.001). This pattern was statistically significant in both the 19–39-year age group (*p* = 0.033) and 40–64-year age group (*p* = 0.001).

Similarly, the prevalence of diabetes was higher in lower-income households than in higher-income households. Participants with a monthly household income of <3.0 million KRW had a prevalence of 13.7%, which was significantly higher than that in the ≥7.0 million KRW group (5.9%, *p* < 0.001). This income-related difference was particularly evident among those aged 40–64 years (*p* = 0.003). Marital status was not significantly associated with the prevalence of diabetes (*p* = 0.386).

Overall, the difference in prevalence between current smokers and nonsmokers was not statistically significant; however, within the 40–64-year age group, the prevalence of diabetes was higher among current smokers (15.0%) than among nonsmokers (9.7%, *p* = 0.020). The prevalence of diabetes was slightly higher in high-risk drinkers (11.7%) than in non–high-risk drinkers (8.7%), although this difference was not statistically significant (*p* = 0.283).

### 3.3. Age-Specific Mean Concentrations of Urinary Phthalate Metabolites Among Participants with Diabetes

Among participants with DM, statistically significant differences in urinary concentrations were observed for seven phthalate metabolites, excluding MCPP (MEHHP, MEOHP, MnBP, MECPP, MBzP, MEP, and MMP; all *p* < 0.001) (Table 3). The highest geometric mean (GM) concentrations were observed for MnBP (28.31 μg/L), followed by MECPP (23.58 μg/L), and MEHHP (17.91 μg/L).

In age-stratified analyses, most phthalate metabolites exhibited the highest concentrations among participants aged ≥65 years with diabetes. For example, MECPP, which showed the second highest GM overall, had a GM concentration of 27.76 μg/L (95% confidence interval (CI), 23.71–32.51) in the ≥65-year DM group, which was higher than that observed in the 40–64-year age group (21.97 μg/L) and the 19–39-year age group (18.17 μg/L) (*p* < 0.001). MCPP was the only metabolite that did not show a statistically significant difference across the overall DM group (*p* = 0.664); however, in age-stratified analyses, MCPP concentrations significantly differed across age groups, with the highest levels observed among participants aged ≥65 years (*p* < 0.001).

### 3.4. Interaction Between Phthalate Metabolites and Age Groups

Interaction analyses examining the associations between urinary phthalate metabolites (categorized by tertiles) and diabetes status identified statistically significant interaction effects with age groups for MnBP (*p* < 0.001), MCPP (*p* = 0.005), and MEP (*p* = 0.038). These findings indicate that the magnitude of the associations between these phthalate metabolites and diabetes status varied significantly by age group, suggesting life-course differences in metabolic vulnerability. The specific effect size estimates (odds ratios) and their 95% confidence intervals for each interaction are presented in Table 4.

### 3.5. Diabetes Prevalence According to Tertiles of Phthalate Exposure

Based on the interaction tests, MnBP, MCPP, and MEP were selected for subsequent age-stratified analyses. Each metabolite was categorized into tertiles, and complex survey-weighted logistic regression models were fitted within each age group to estimate the odds of diabetes (prevalent DM) across exposure levels (Table 5 and Table 6). Statistically significant associations were observed for MnBP, MCPP, and MEP; and for some age groups, the magnitude and statistical significance differed between the unadjusted and adjusted models.

#### 3.5.1. Mono-n-Butyl Phthalate (MnBP)

In the 19–39-year age group, the highest exposure tertile (T3) was associated with a significantly higher odds of diabetes compared with the lowest tertile (T1) in the unadjusted model (odds ratio [OR] = 6.56; 95% CI, 1.34–32.13). This association remained statistically significant after adjustment, with a larger effect estimate (OR = 10.04; 95% CI, 1.46–69.05; *p* < 0.001).

In the ≥65-year age group, diabetes prevalence differed significantly across MnBP tertiles (unadjusted *p* = 0.006, adjusted *p* = 0.003). Relative to T1, both T2 and T3 showed increased odds of diabetes in the unadjusted model (T2: OR = 2.46, 95% CI: 1.44–4.19; T3: OR = 1.58, 95% CI: 0.83–2.98) and in the adjusted model (T2: OR = 2.52, 95% CI: 1.50–4.22; T3: OR = 1.61, 95% CI: 0.85–3.04).

#### 3.5.2. Mono(3-Carboxypropyl) Phthalate (MCPP)

In the 19–39-year age group, diabetes prevalence significantly differed across tertiles of MCPP exposure (unadjusted *p* < 0.001, adjusted *p* < 0.001). Compared with T1, the highest exposure tertile (T3) showed an elevated odds of diabetes, although the confidence intervals were wide (unadjusted: OR = 3.32, 95% CI: 0.59–18.81; adjusted: OR = 3.89, 95% CI: 0.72–21.01).

#### 3.5.3. Monoethyl Phthalate (MEP)

In the 19–39-year age group, both the unadjusted and adjusted models indicated statistically significant differences across MEP tertiles, with an overall pattern suggestive of reduced odds of diabetes in T2 and T3 relative to T1. However, the effect estimates were small, indicating markedly lower odds than those of the reference group.

In the 40–64-year age group, the association between MEP tertiles and diabetes was not statistically significant in the unadjusted model. After adjustment, participants in the highest tertile (T3) had significantly higher odds of diabetes compared with those in T1 (OR = 1.68; 95% CI, 1.07–2.63; *p* = 0.039). In the ≥65-year age group, the unadjusted comparison between T3 and T1 did not reach statistical significance (OR = 1.89; 95% CI, 1.02–3.53; *p* = 0.099). In the adjusted model, T3 was associated with increased odds of diabetes (OR = 2.07; 95% CI, 1.16–3.71; *p* = 0.027).

## 4. Discussion

This study used nationally representative biomonitoring data from the 4th KoNEHS (2018–2020) to investigate associations between urinary phthalate metabolite concentrations and the prevalence of diabetes among Korean adults. In contrast to prior cross-sectional analyses that typically treated the adult population as a single group, we performed age-stratified analyses across three life-course stages—young adults (19–39 years), middle-aged adults (40–64 years), and older adults (≥65 years)—to reflect heterogeneity in metabolic profiles and exposure pathways across age groups.

In the overall population, GM concentrations of multiple urinary phthalate metabolites (MEHHP, MEOHP, MnBP, MECPP, MBzP, MEP, and MMP) were statistically significant. This pattern aligns with previous evidence suggesting that exposure to these metabolites may be associated with diabetes risk [4,26,27]. A growing body of epidemiological research has repeatedly reported associations between urinary phthalate metabolites and diabetes or markers of insulin resistance. For example, NHANES-based studies have documented positive associations for several metabolites and have further raised the possibility of non-monotonic exposure–response patterns [10]. In addition, recent meta-analytic evidence indicates that phthalate exposure may be related to an increased overall diabetes risk [9]. Consistent with our results, analyses of adult participants in KoNEHS Cycle 3 (2015–2017) also reported a higher prevalence of diabetes among individuals with higher urinary concentrations of phthalate metabolites [23].

In contrast, some prior studies have reported findings that are not fully consistent with our results. In a 6-year prospective cohort study of women examining whether phthalate exposure increased incident diabetes, the associations differed by race/ethnicity. Specifically, among White women, a two-fold increase in several phthalate metabolites—including MiBP, MBzP, mono-carboxyoctyl phthalate (MCOP), mono-carboxyisononyl phthalate, and MCPP—was associated with a 30–63% higher diabetes incidence, whereas no statistically significant associations were observed among Black or Asian women. The authors therefore suggested that the relationship between phthalate metabolites and diabetes incidence may lack consistency across racial/ethnic groups, highlighting the need for further research [28]. In addition, a meta-analysis of seven studies evaluating the association between phthalate exposure and diabetes risk reported that exposure to MMP, MnBP, MiBP, MCPP, and DEHP metabolites was associated with a higher risk of diabetes, whereas MEP, MBzP, and mono(2-ethylhexyl) phthalate (MEHP) were not significantly associated with diabetes risk [9].

Potential biological mechanisms linking phthalate exposure to diabetes have been extensively explored in experimental and epidemiological studies [23,29]. Specifically, phthalates and their metabolites may act as ligands for peroxisome proliferator-activated receptors (PPARs), particularly PPAR-γ, which plays a critical role in glucose and lipid metabolism [26]. Disruption of this nuclear receptor signaling may impair adipogenesis and reduce systemic insulin sensitivity. Furthermore, prior studies have suggested that phthalate exposure can induce oxidative stress—characterized by increased levels of 8-hydroxy-2′-deoxyguanosine and malondialdehyde—and that such oxidative stress is significantly associated with increased insulin resistance (HOMA-IR) [30,31]. Oxidative damage may interfere with insulin signaling pathways, thereby compromising glucose metabolism. Because elevated HOMA-IR is a key pathophysiological precursor to T2D, increases in insulin resistance may translate into a higher clinical risk of developing diabetes mellitus.

Importantly, urinary metabolite concentrations significantly differed across age groups among participants with diabetes. Building on these findings, we further examined age-specific susceptibility to MnBP, MCPP, and MEP, which demonstrated statistically significant interaction effects. In age-stratified multivariate models adjusted for socioeconomic and lifestyle factors, the patterns of association substantially varied according to age group. Among young adults (19–39 years), higher exposure to MnBP and MCPP was associated with a markedly increased risk of diabetes. In middle-aged adults (40–64 years), MEP was positively associated with diabetes prevalence, whereas in older adults (≥65 years), MnBP and MEP showed significant positive associations. Overall, these findings support the presence of life-course heterogeneity in the phthalate–diabetes association and suggest that the relevant metabolites and the magnitude of association may differ across age strata.

MEP exhibited clear age-dependent heterogeneity; the ORs were below 1.0 in the young adult group, and they exceeded 1.0 in the middle-aged and older adult groups. This pattern suggests the presence of differential vulnerability and/or exposure by age, whereby the same chemical exposure may yield different metabolic consequences depending on the life course [4,32]. The elevated odds of diabetes observed in the high-MEP exposure group among middle-aged and older adults may be partly attributable to age-related changes in renal function, body composition, and hormonal milieu and increased inflammatory and oxidative stress burden, which can amplify metabolic strain even under comparable exposure levels [33,34]. In addition, because the baseline prevalence of diabetes is substantially higher in middle-aged and older populations, a similar relative shift in exposure may translate into a greater absolute disease burden at the population level [35].

In contrast, the finding of ORs < 1.0 in young adults should not be interpreted as evidence of a protective effect of MEP. Careful interpretation is warranted because the number of diabetes cases in this age group is likely to be significantly small, which can yield unstable estimates and wide uncertainty. Moreover, in cross-sectional settings, diabetes defined by a self-reported physician diagnosis typically reflects a state accompanied by medical management and lifestyle modifications [36]. If a small subset of young adults with diabetes disproportionately avoids specific consumer products or personal care items that contribute to MEP exposure, the observed exposure–outcome association may be distorted through reverse causation or behavior-related confounding. Therefore, the apparent age-specific directionality of MEP should be re-evaluated in future studies with larger numbers of young-onset diabetes cases and more comprehensive adjustments for potential confounders to confirm whether the observed pattern persists.

### 4.1. Diabetes and MnBP/MCPP in Young Adults (19–39 Years): Environmental Risk Factors for Early-Onset Type 2 Diabetes

The most notable finding of this study was the pronounced association observed in the 19–39-year age group, in which individuals in the highest tertile of MnBP exposure (T3) showed approximately tenfold higher odds of developing diabetes than those in the lowest tertile (T1). This magnitude represents a strong association that extends beyond statistical significance and appears to be a distinctive pattern in young adults, as similarly large effects were not observed in the middle-aged (40–64 years) or older (≥65 years) groups. Although the elevated odds in the high-exposure group were already evident in the unadjusted model, the association persisted and increased in magnitude in the adjusted model. In addition, MCPP warrants particular attention because, despite showing no significant difference in the overall pooled analysis across all adults, it emerged as a meaningful predictor of diabetes, specifically among young adults in the adjusted model. Taken together, these findings suggest that selected phthalate metabolites may be differentially relevant to diabetes risk within younger age strata.

These age-specific patterns may be interpreted in the broader epidemiological context of the increasing incidence of early-onset T2D among children, adolescents, and young adults [2]. Increasing attention has been directed toward EDCs, often described as environmental obesogens, as potential contributors to metabolic dysregulation beyond the traditional behavioral risk factors [37]. Our findings provide additional population-based evidence supporting the hypothesis that specific environmental chemicals, such as MnBP and MCPP, may contribute to the pathophysiology of early-onset T2D in young adults.

Both MnBP and MCPP are metabolites of plasticizers and are associated with exposure sources, such as personal care products (PCPs) (e.g., cosmetics, perfumes, and hair sprays) and food contact materials, including packaging commonly used for fast and convenience foods [5,6]. In the Republic of Korea, individuals in their 20s and 30s tend to have a relatively high use of PCPs for appearance-related practices and a higher reliance on delivered meals and processed foods than older age groups. Notably, the KoNEHS Cycle 4 data collection period (2018–2020) overlapped with the onset of the coronavirus disease 2019 pandemic in 2020, during which increased consumption of delivered foods may have further intensified plastic-related exposure. Phthalates can migrate from plastic containers to food, particularly when hot foods are stored in plastic packaging or when foods are heated in plastic containers using microwaves, potentially increasing dietary exposure. Such patterns may contribute to sustained higher exposure to MnBP and MCPP in everyday settings [5].

Previous studies have supported these exposure pathways. Dose–response associations have been reported in which higher fast-food intake was associated with higher urinary concentrations of MCPP and metabolites of diisononyl phthalate [19]. Korean studies have also reported higher phthalate metabolite concentrations among individuals who frequently consume convenience store lunch boxes and delivered food [38]. Furthermore, urinary MnBP concentrations are higher in women than in men and higher in younger age groups than in older age groups [6]. In addition, previous epidemiological findings showing positive associations between MnBP/MCPP concentrations and fasting glucose levels or insulin resistance markers, such as Homeostatic Model Assessment of Insulin Resistance [10], are consistent with the overall direction of our results, supporting the plausibility of MnBP and MCPP as environmentally relevant correlates of metabolic risk in young adults.

### 4.2. Diabetes and MEP in Older Adults (≥65 Years): The Potential Role of Reverse Causality

In the analysis of adults aged ≥65 years, urinary MEP concentrations showed a statistically significant positive association with diabetes prevalence. In the fully adjusted model, participants in the highest exposure tertile (T3) exhibited approximately twofold higher odds of developing diabetes than those in the lowest tertile (T1) (OR = 2.07, *p* = 0.027). This finding is consistent with results from the Prospective Investigation of the Vasculature in Uppsala Seniors study conducted among Swedish adults aged ≥70 years, which also reported an association between MEP exposure and diabetes [26]. Nevertheless, interpretation of the potentially diabetogenic effect of MEP requires careful consideration of reverse causality.

Diethyl phthalate (DEP), the parent compound of MEP, is widely used as a plasticizer in enteric coatings that allow medications to resist gastric acid and dissolve in the intestine. Older adults with diabetes frequently engage in polypharmacy, including the long-term use of glucose-lowering agents, antihypertensive medications, and aspirin. Previous research has demonstrated that urinary concentrations of DBP and DEP metabolites can be several orders of magnitude higher in patients taking specific enteric-coated medications than in the general population [39]. Thus, elevated urinary MEP concentrations may not necessarily precede diabetes onset but may instead reflect increased exposure resulting from pharmacological treatment for diabetes and its comorbidities.

Given the cross-sectional nature of the present study and the lack of detailed information on medication use, the possibility that higher MEP levels arise as a consequence of medical treatment rather than as a causal factor cannot be excluded. Despite these limitations, our findings raise important concerns regarding iatrogenic phthalate exposure as an additional health burden in older adults. This consideration is particularly relevant in light of evidence suggesting that phthalates may exacerbate insulin resistance [26,31] and that phthalate exposure among individuals with diabetes is associated with increased cardiovascular mortality [25,27]. Taken together, these findings underscore the need to re-evaluate the safety of pharmaceutical excipients used in chronic disease management, especially among vulnerable older populations with high medication burdens. Accordingly, the higher urinary metabolite concentrations observed among participants with diabetes in this study may reflect both (1) the possibility that phthalate exposure contributes to metabolic dysregulation and (2) the possibility that diabetes management and comorbidity-related care alter exposure sources and metabolism/excretion patterns. Therefore, the directionality of this association cannot be determined from cross-sectional data alone.

### 4.3. Socioeconomic Health Inequalities and the Need for an Environmental Health Equity Perspective

In the descriptive analyses, the prevalence of diabetes markedly differed according to socioeconomic status (SES), including educational level and household income. A clear social gradient was observed in the overall sample, with a higher prevalence of diabetes among individuals with lower educational attainment and household income. In age-stratified analyses, educational disparities were statistically significant among young and middle-aged adults but not among older adults, whereas income-related disparities were significant only in the middle-aged group. These findings suggest that socioeconomic inequalities in diabetes do not manifest uniformly across the life course but may vary in magnitude depending on age and social context.

Middle adulthood may represent a critical period during which multiple Social Determinants of Health accumulate, including occupational demands, caregiving responsibilities, constraints on time and resources for health-promoting behaviors, and differential access to continuous healthcare services [40]. Such cumulative exposures may contribute to disparities in the risk and management of chronic diseases, including diabetes [41]. Consistent with this interpretation, the WHO has emphasized that structural interventions targeting upstream social determinants are central to reducing health inequities [42].

Beyond conventional interpretations of SES as a source of differential exposure, the association between phthalate exposure and diabetes highlighted in this study calls for consideration of differential vulnerability. Even at comparable levels of environmental exposure, individuals in socially disadvantaged conditions may experience a greater metabolic burden due to co-occurring factors, such as poor nutrition, substandard housing, occupational stress, psychosocial strain, and limited access to health care. This conceptual framework is central to an environmental health equity perspective [41]. Empirical evidence indicates that residential factors, including indoor building materials, such as vinyl flooring, can act as sources of phthalate exposure [7], and housing conditions in lower-income settings may contribute to health disparities through increased environmental exposure [43].

Furthermore, growing evidence links dietary patterns and food environments to the urinary concentrations of specific phthalate metabolites, including MEP, MCPP, and MCOP [19,44]. Therefore, socioeconomic constraints that shape food choice and consumption patterns may mediate a “double burden” in which disadvantaged populations face both higher environmental exposures and greater susceptibility to metabolic disorders. This intersection underscores the importance of integrating socioeconomic context into environmental epidemiology.

Accordingly, future studies should move beyond treating SES merely as an adjustment variable and instead (1) formally test effect modification by SES in the phthalate–diabetes association and (2) incorporate detailed data on exposure pathways, such as housing characteristics, food environments, and personal care product use, to more precisely identify points at which environmental exposure inequality and metabolic disease inequality intersect [41]. From a policy perspective, multilevel interventions are warranted, including targeted health education on phthalate-related risks for vulnerable populations, improved transparency and readability of product and packaging information, enhancements to housing environments, and policies aimed at increasing access to healthy foods [42].

### 4.4. Public Health Implications: Age-Tailored Exposure Reduction and Surveillance Strategies

The age-specific heterogeneity observed in the phthalate–diabetes association in this study suggests that both dominant behavioral sources of exposure and biological susceptibility may differ across population subgroups. Accordingly, age-tailored exposure reduction strategies may improve public health efficiency by targeting the most relevant exposure contexts for each age group. Recent evidence indicates that higher consumption of ultra-processed foods may contribute to elevated phthalate biomarkers through food processing and packaging pathways and that socioeconomic conditions may exacerbate exposure disparities via these mechanisms [45]. In addition, research discussing phthalate exposure alongside oxidative stress-related markers in T2D supports the need for surveillance frameworks that move beyond single-chemical approaches and incorporate mixed exposure together with biological pathways, such as oxidative stress and endocrine disruption [4].

Based on these findings, we propose the following policies and practical priorities. First, to reduce phthalate exposure among young adults, interventions should align with media-use patterns and health behaviors. Modern strategies include the use of mobile health applications that provide data-driven, personalized feedback. In parallel, public communication campaigns can leverage social media to disseminate “No-Chemi” lifestyle messaging in formats familiar to younger audiences. Moreover, regulatory standards for phthalate migration from plastics commonly used in food delivery containers, particularly materials such as polyvinyl chloride and polystyrene, should be strengthened to reflect the high-temperature conditions and frequent contact with fatty foods, which may increase the likelihood of chemical migration.

Second, for older adults in whom iatrogenic exposure is strongly suspected, pharmaceutical labeling policies require clear disclosure when formulations contain excipients of concern, such as DEP or DBP, which have potential endocrine-disrupting properties. In clinical practice, systems should be established to enable prescribers and pharmacists to readily verify phthalate-containing excipients and consider appropriate alternatives when available. In addition, policy incentives can encourage the pharmaceutical industry to replace phthalates with safer plant-derived polymers or nontoxic plasticizers, consistent with “Green Pharmacy” approaches aimed at reducing hazardous excipients in chronic disease management [46].

Third, from the perspectives of socioeconomic health equity and environmental justice, multilevel interventions should be implemented to reduce the combined burden of social disadvantage and environmental exposure. For example, expansion of food voucher programs that facilitate access to fresh fruits and vegetables and other “phthalate-free” food options may be particularly beneficial in low-income communities and households of older adults. Housing improvement initiatives should prioritize and support the use of phthalate-free building materials in publicly funded renovation programs, including flooring and wallpaper. Additionally, tailored environmental health education programs should be implemented to strengthen health literacy among socially vulnerable groups.

### 4.5. Limitations

This study has some limitations. First, the cross-sectional design precludes causal inference, and reverse causation cannot be ruled out, particularly if the diagnosis of diabetes leads to changes in lifestyle or consumer product use that alter exposure patterns. Second, phthalate metabolites were measured using a single-spot urine sample; given the short biological half-lives of many phthalates, these measures may not fully capture long-term exposure. Third, although we attempted to address lifestyle factors by including smoking status and high-risk drinking as covariates (as shown in Table 2), other important confounders, including detailed dietary patterns, individual oxidative stress levels, and precise physical activity measures, were not fully incorporated due to data constraints in the KoNEHS 4th cycle (2018–2020). This limits our ability to precisely identify exposure sources and disentangle potential iatrogenic pathways. Fourth, the KoNEHS data did not distinguish between Type 1 and Type 2 diabetes. However, as the majority of adult-onset diabetes cases in the Korean population are Type 2, the inclusion of Type 1 cases is expected to have a negligible impact on the overall findings. Fifth, because the number of diabetes cases among young adults was small, estimates may be unstable, and some extreme OR values should be interpreted cautiously. Future studies with prospective cohort designs, clinical diagnostic criteria, and rich covariate information are required to validate these findings.

## 5. Conclusions

Using nationally representative biomonitoring data from the 4th Korean National Environmental Health Survey (KoNEHS, 2018–2020), this cross-sectional study suggests that associations between urinary phthalate metabolites and diabetes among Korean adults may vary across age strata. In analyses accounting for socioeconomic and health-behavior factors, selected metabolites—MnBP and MCPP among young adults and MEP among older adults—exhibited differing age-stratified patterns. These findings may indicate that environmental interventions could be relevant for preventing early-onset diabetes in younger populations, while the observed association with MEP in older adults raises the possibility of iatrogenic exposure pathways related to medication use.

Nevertheless, because causal inference is inherently limited in a cross-sectional design, longitudinal confirmation is warranted to verify age-specific heterogeneity in the phthalate–diabetes relationship using more precise exposure assessment and outcome ascertainment. Despite these limitations, our results underscore that effective diabetes prevention and management may benefit from policy-level consideration of broader environmental health strategies aimed at monitoring and reducing population-level exposure to hazardous environmental chemicals embedded in everyday life.

## Figures and Tables

**Table 1:** General characteristics.

		All (Age < 18)		(19–39)		(40–64)		(65 ≤ Age)	
		N	%	N	%	N	%	N	%
Total		4239	(100.0)	980	(35.3)	2247	(46.3)	1012	(18.4)
Sex									
	Male	1889	(49.8)	442	(52.6)	951	(49.9)	496	(44.3)
	Female	2350	(50.2)	538	(47.4)	1296	(50.1)	516	(55.7)
Education									
	≥College	1844	(51.6)	814	(81.9)	907	(44.3)	123	(12.0)
	High school	1293	(29.5)	159	(17.4)	893	(41.0)	241	(23.7)
	≤Middle	1102	(18.9)	7	(0.7)	447	(14.8)	648	(64.3)
Household income (≈USD/month)									
	≥7 M KRW (≥5.2 K)	453	(11.7)	135	(14.0)	285	(12.9)	33	(3.5)
	5–7 M KRW (3.7–5.2 K)	682	(18.2)	230	(24.4)	420	(19.1)	32	(2.7)
	3–5 M KRW (2.2–3.7 K)	1137	(29.5)	329	(32.9)	695	(33.0)	114	(12.1)
	<3 M KRW (<2.2 K)	1931	(40.6)	272	(26.5)	835	(34.3)	824	(81.0)
Marital status									
	No	1091	(33.1)	504	(61.3)	317	(12.8)	270	(30.4)
	Yes	3148	(66.9)	476	(38.7)	1930	(87.2)	742	(69.6)
Current smoking									
	No	3548	(81.3)	796	(80.2)	1859	(78.9)	893	(89.4)
	Yes	691	(18.7)	184	(19.8)	388	(21.1)	119	(10.6)
High-risk drinking									
	No	3828	(89.9)	884	(90.5)	1997	(87.1)	947	(95.8)
	Yes	411	(10.1)	96	(9.5)	250	(12.9)	65	(4.2)
Diabetes mellitus									
	Non-DM	2663	(90.9)	618	(97.9)	1462	(89.1)	583	(81.4)
	DM	325	(9.1)	13	(2.1)	179	(10.9)	133	(18.6)

All values were weighted to account for the complex survey design (N = unweighted sample size; % = weighted proportion adjusted for the complex survey design).

**Table 2:** Cross-tabulation of general characteristics according to diabetes status.

		All (Age < 18)					(19–39)					(40–64)					(65 ≤ Age)					*p* *
		Non-DM		DM			Non-DM		DM			Non-DM		DM			Non-DM		DM			
		N	(%)	N	(%)	*p*	N	(%)	N	(%)	*p*	N	(%)	N	(%)	*p*	N	(%)	N	(%)	*p*	
Total		2663	(90.9)	325	(9.1)		618	(98.3)	13	(1.7)		1462	(89.2)	179	(10.8)		583	(83.5)	133	(16.5)		
Sex						0.002					0.102					<0.001					0.672	0.003
	Male	1127	(88.5)	170	(11.5)		266	(97.4)	7	(2.6)		578	(84.7)	95	(15.3)		283	(82.5)	68	(17.5)		
	Female	1536	(93.3)	155	(6.7)		352	(99.3)	6	(0.7)		884	(93.4)	84	(6.6)		300	(84.3)	65	(15.7)		
Education						<0.001					0.033					0.001					0.168	<0.001
	≥College	1211	(96.2)	73	(3.8)		532	(99.0)	9	(1.0)		605	(93.7)	47	(6.3)		74	(89.5)	17	(10.5)		
	High school	778	(87.2)	115	(12.8)		83	(94.2)	4	(5.8)		570	(87.8)	74	(12.2)		125	(76.7)	37	(23.3)		
	≤Middle	674	(82.7)	137	(17.3)		3	(100.0)	0	(0.0)		287	(79.7)	58	(20.3)		384	(84.5)	79	(15.5)		
Household income						<0.001					0.574					0.003					0.912	<0.001
	≥7 M KRW	301	(94.1)	25	(5.9)		81	(100.0)	0	(0.0)		200	(91.8)	19	(8.2)		20	(84.5)	6	(15.5)		
	5–7 M KRW	482	(95.8)	27	(4.2)		155	(99.0)	2	(1.0)		306	(94.2)	24	(5.8)		21	(79.4)	1	(20.6)		
	3–5 M KRW	734	(92.7)	65	(7.3)		229	(98.0)	3	(2.0)		438	(90.7)	44	(9.3)		67	(80.8)	18	(19.2)		
	<3 M KRW	1143	(86.3)	208	(13.7)		153	(97.1)	8	(2.9)		518	(83.7)	92	(16.3)		472	(84.0)	108	(16.0)		
Marital status						0.386					0.587					0.06					0.520	0.386
	No	629	(92.1)	77	(7.9)		292	(98.0)	6	(2.0)		186	(84.6)	33	(15.4)		151	(81.6)	38	(18.4)		
	Yes	2034	(90.5)	248	(9.5)		326	(98.7)	7	(1.3)		1276	(89.8)	146	(10.2)		432	(84.3)	95	(15.7)		
Current smoking						0.129					0.287					0.02					0.304	0.129
	No	2261	(91.6)	262	(8.4)		511	(98.7)	7	(1.3)		1229	(90.3)	137	(9.7)		521	(84.3)	118	(15.7)		
	Yes	402	(88.1)	63	(11.9)		107	(96.9)	6	(3.1)		233	(85.0)	42	(15.0)		62	(76.3)	15	(23.7)		
High-risk drinking						0.283					0.433					0.149					0.389	0.283
	No	2391	(91.3)	282	(8.7)		553	(98.1)	13	(1.9)		1297	(90.1)	146	(9.9)		541	(83.8)	123	(16.2)		
	Yes	272	(88.3)	43	(11.7)		65	(100.0)	0	(0.0)		165	(84.2)	33	(15.8)		42	(76.9)	10	(23.1)		

* *p*-values represent comparisons between non-diabetes and diabetes groups.

**Table 3:** Mean concentrations of phthalate metabolites among individuals with diabetes according to age group.

	All (Age < 18)		*p*	(19–39)		(40–64)		(65 ≤ Age)		*p*
	GM ^1^	(95% CI)		GM	(95% CI)	GM	(95% CI)	GM	(95% CI)	
MEHHP	17.91	(16.47–19.47)	<0.001	11.33	(7.57–16.96)	17.10	(14.91–19.60)	20.89	(17.79–24.53)	<0.001
MEOHP	9.83	(8.89–10.86)	<0.001	6.43	(4.04–10.22)	9.29	(7.84–11.01)	11.60	(9.40–14.32)	<0.001
MnBP	28.31	(23.74–33.74)	<0.001	32.13	(19.73–52.34)	26.29	(21.20–32.61)	31.45	(24.53–40.33)	<0.001
MECPP	23.58	(21.55–25.79)	<0.001	18.17	(12.78–25.84)	21.97	(19.08–25.29)	27.76	(23.71–32.51)	<0.001
MBzP	1.32	(1.12–1.56)	<0.001	0.57	(0.30–1.11)	1.25	(0.99–1.57)	1.67	(1.29–2.15)	<0.001
MCPP	0.43	(0.35–0.54)	0.664	0.44	(0.27–0.74)	0.40	(0.32–0.50)	0.50	(0.38–0.66)	<0.001
MEP	8.16	(4.74–14.06)	0.027	3.15	(1.10–9.02)	6.39	(3.64–11.24)	14.49	(7.01–29.97)	0.002
MMP	4.25	(3.77–4.79)	<0.001	4.94	(3.00–8.13)	3.58	(3.05–4.20)	5.56	(4.76–6.49)	<0.001

^1^ GM, geometric mean.

**Table 4:** Interaction effects of phthalate metabolites and age groups on the prevalence of DM.

Metabolite	Interaction Terms		OR	(95% CI)	*p*	Interaction *p* *
	Age	Tertile				
(Ref)	19–39	T1				
MEHHP	40–64	T2	0.82	(0.12–5.61)	0.837	0.358
		T3	5.41	(0.67–43.39)	0.114	
	≥65	T2	0.40	(0.05–3.37)	0.404	
		T3	2.45	(0.27–22.44)	0.430	
MEOHP	40–64	T2	1.02	(0.15–6.81)	0.983	0.109
		T3	8.01	(0.87–73.80)	0.068	
	≥65	T2	0.33	(0.04–2.60)	0.295	
		T3	2.80	(0.26–30.26)	0.399	
MnBP	40–64	T2	27.90	(2.87–270.76)	0.005	<0.001
		T3	0.20	(0.04–1.05)	0.059	
	≥65	T2	62.68	(6.58–597.37)	<0.001	
		T3	0.19	(0.03–1.15)	0.073	
MECPP	40–64	T2	0.98	(0.16–6.10)	0.981	0.902
		T3	0.46	(0.04–4.75)	0.512	
	≥65	T2	0.63	(0.09–4.52)	0.648	
		T3	0.32	(0.03–3.80)	0.366	
MBzP	40–64	T2	0.75	(0.10–5.62)	0.776	0.723
		T3	2.65	(0.38–18.66)	0.329	
	≥65	T2	0.69	(0.08–6.03)	0.741	
		T3	1.91	(0.22–16.64)	0.560	
MCPP	40–64	T2	14.33	(2.12–97.01)	0.007	0.005
		T3	0.24	(0.04–1.53)	0.134	
	≥65	T2	25.50	(3.71–175.21)	0.001	
		T3	0.27	(0.04–1.82)	0.180	
MEP	40–64	T2	42.78	(3.66–500.73)	0.003	0.023
		T3	2.78	(0.51–15.20)	0.239	
	≥65	T2	77.38	(6.07–986.27)	0.001	
		T3	4.31	(0.69–26.71)	0.119	
MMP	40–64	T2	0.37	(0.06–2.21)	0.276	0.069
		T3	0.16	(0.02–1.62)	0.124	
	≥65	T2	1.43	(0.25–8.21)	0.692	
		T3	0.52	(0.05–5.30)	0.584	

* Interaction *p*-values were obtained from the joint Wald test of the age group × metabolite tertile interaction terms in survey-weighted logistic regression models. The reference categories were the lowest tertile (Tertile 1) and the 19–39 age group. All models accounted for the complex sampling design and were adjusted for sex, education level, household income, marital status, current smoking, and high-risk drinking.

**Table 5:** Risk of DM according to tertiles of phthalate metabolite exposure (unadjusted model).

		(19–39)				(40–64)				(65 ≤ Age)			
		OR	(95% CI)	*p*	*p*c	OR	(95% CI)	*p*	*p*c	OR	(95% CI)	*p*	*p*c
MnBP	T1	Ref		<0.001	<0.008	Ref		0.489	1.000	Ref		0.006	0.048
	T2	0.04	(0.00–0.39)			0.99	(0.61–1.60)			2.46	(1.44–4.19)		
	T3	6.56	(1.34–32.13)			1.27	(0.81–1.99)			1.58	(0.83–2.98)		
MCPP	T1	Ref		<0.001	<0.008	Ref		0.246	1.000	Ref		0.737	1.000
	T2	0.05	(0.01–0.28)			0.62	(0.30–1.30)			1.12	(0.58–2.19)		
	T3	3.32	(0.59–18.81)			0.92	(0.50–1.71)			1.28	(0.69–2.39)		
MEP	T1	Ref		0.008	0.064	Ref		0.23	1.000	Ref		0.099	0.792
	T2	0.02	(0.00–0.24)			0.73	(0.41–1.30)			1.32	(0.62–2.81)		
	T3	0.52	(0.09–2.83)			1.20	(0.76–1.92)			1.89	(1.02–3.53)		

*p*: nominal significance value; *p*c: Bonferroni-corrected *p*-value (*p* × 8). To account for multiple comparisons across eight phthalate metabolites, statistical significance was defined at *p*c < 0.05 (equivalent to a nominal *p* < 0.00625).

**Table 6:** Risk of DM according to tertiles of phthalate metabolite exposure (adjusted model).

		(19–39)				(40–64)				(65 ≤ Age)			
		OR	(95% CI)	*p*	*p*c	OR	(95% CI)	*p*	*p*c	OR	(95% CI)	*p*	*p*c
MnBP	T1	Ref		<0.001	<0.008	Ref		0.347	1.000	Ref		0.003	0.024
	T2	0.06	(0.01–0.61)			0.99	(0.61–1.61)			2.52	(1.50–4.22)		
	T3	10.04	(1.46–69.05)			1.40	(0.83–2.36)			1.61	(0.85–3.04)		
MCPP	T1	Ref		<0.001	<0.008	Ref		0.199	1.000	Ref		0.665	1.000
	T2	0.06	(0.01–0.50)			0.70	(0.37–1.34)			1.17	(0.62–2.22)		
	T3	3.89	(0.72–21.01)			1.12	(0.62–2.03)			1.30	(0.72–2.36)		
MEP	T1	Ref		0.017	0.136	Ref		0.039	0.312	Ref		0.027	0.216
	T2	0.03	(0.00–0.35)			0.92	(0.53–1.59)			1.34	(0.64–2.78)		
	T3	0.68	(0.10–4.55)			1.68	(1.07–2.63)			2.07	(1.16–3.71)		

*p*: nominal significance value; *p*c: Bonferroni-corrected *p*-value (*p* × 8). To account for multiple comparisons across eight phthalate metabolites, statistical significance was defined at *p*c < 0.05 (equivalent to a nominal *p* < 0.00625). Models were adjusted for sex, educational level, household income, marital status, current smoking status, and high-risk drinking status.

## Data Availability

The data analyzed in this study were obtained from the Korean National Environmental Health Survey (KoNEHS) Cycle 4 (2018–2020). These data are not publicly available due to privacy and ethical restrictions but can be accessed upon reasonable request and approval from the National Institute of Environmental Research (NIER), in accordance with official data use guidelines.

## Abbreviations

The following abbreviations are used in this manuscript:

DM	Diabetes mellitus
T2D	Type 2 diabetes
NHANES	National Health and Nutrition Examination Survey
KoNEHS	Korean National Environmental Health Survey
NIER	National Institute of Environmental Research
MEHHP	Mono(2-ethyl-5-hydroxyhexyl) phthalate
MEOHP	Mono(2-ethyl-5-oxohexyl) phthalate
MnBP	Mono-n-butyl phthalate
MECPP	Mono(2-ethyl-5-carboxypentyl) phthalate
MBzP	Monobenzyl phthalate
MCPP	Mono(3-carboxypropyl) phthalate
MEP	Monoethyl phthalate
MMP	Monomethyl phthalate
GM	Geometric mean
OR	Odds ratio
CI	Confidence interval

## References

[1] World Health Organization (WHO) Diabetes. https://www.who.int/news-room/fact-sheets/detail/diabetes.

[2] Wagenknecht L.E., Lawrence J.M., Isom S., Jensen E.T., Dabelea D., D’Liese A., Dolan L.M., Shah A.S., Bellatorre A., Sauder K. (2023). Trends in incidence of youth-onset type 1 and type 2 diabetes, 2002–2018: Results from the US population-based SEARCH for diabetes in youth study. Lancet Diabetes Endocrinol..

[3] Makhubela S.D., Kgopa A.H., Mokgotho M.P., Shai L.J. (2024). Di(2-ethylhexyl)phthalate and type 2 diabetes. Environ. Sci. Adv..

[4] Mariana M., Cairrao E. (2023). The relationship between phthalates and diabetes: A review. Metabolites.

[5] FDA (U.S. Food and Drug Administration) Phthalates in Cosmetics. https://www.fda.gov/cosmetics/cosmetic-ingredients/phthalates-cosmetics.

[6] Fišerová P.S., Melymuk L., Komprdová K., Domínguez-Romero E., Scheringer M., Kohoutek J., Přibylová P., Andrysíková L., Piler P., Koch H.M. (2022). Personal care product use and lifestyle affect phthalate and DINCH metabolite levels in teenagers and young adults. Environ. Res..

[7] Just A.C., Miller R.L., Perzanowski M.S., Rundle A.G., Chen Q., Jung K.H., Hoepner L., Camann D.E., Calafat A.M., Perera F.P. (2015). Vinyl flooring in the home is associated with children’s airborne butylbenzyl phthalate and urinary metabolite concentrations. J. Expo. Sci. Environ. Epidemiol..

[8] Hong S., Kim O.-J., Jung S.K., Jeon H.Y., Kim S., Kil J. (2024). The exposure status of environmental chemicals in South Korea: The Korean National Environmental Health Survey 2018–2020. Toxics.

[9] Zhang H., Ben Y., Han Y., Zhang Y., Li Y., Chen X. (2022). Phthalate exposure and risk of diabetes mellitus: Implications from a systematic review and meta-analysis. Environ. Res..

[10] James-Todd T., Stahlhut R., Meeker J.D., Powell S.-G., Hauser R., Huang T., Rich-Edwards J. (2012). Urinary phthalate metabolite concentrations and diabetes among women in the National Health and Nutrition Examination Survey (NHANES) 2001–2008. Environ. Health Perspect..

[11] Darwish R., Alcibahy Y., Abu-Sharia G., Butler A.E. (2025). β-cell mitochondrial dysfunction: Underlying mechanisms and potential therapeutic strategies. Cells.

[12] Ghanati K., Pourjafar H., Shayali-Gilani P., Akbari N., Tavoosidana G., Zandsalimi F., Abdolhosseini M., Aghaei M., Sadighara P. (2025). The effect of phthalates on insulin secretion of pancreatic beta cells based on cellular studies, a systematic review. Toxicol. Lett..

[13] Hurst C.H., Waxman D.J. (2003). Activation of PPARα and PPARγ by environmental phthalate monoesters. Toxicol. Sci..

[14] Deng T., Zhang Y., Wu Y., Ma P., Duan J., Qin W., Yang X., Chen M. (2018). Dibutyl phthalate exposure aggravates type 2 diabetes by disrupting the insulin-mediated PI3K/AKT signaling pathway. Toxicol. Lett..

[15] Ehrhardt N., Cui J., Dagdeviren S., Saengnipanthkul S., Goodridge H.S., Kim J.K., Lantier L., Guo X., Chen Y.D.I., Raffel L.J. (2019). Adiposity-independent effects of aging on insulin sensitivity and clearance in mice and humans. Obesity.

[16] Ou M.Y., Zhang H., Tan P.C., Zhou S.B., Li Q.F. (2022). Adipose tissue aging: Mechanisms and therapeutic implications. Cell Death Dis..

[17] Zuo L., Prather E.R., Stetskiv M., Garrison D.E., Meade J.R., Peace T.I., Zhou T. (2019). Inflammaging and oxidative stress in human diseases: From molecular mechanisms to novel treatments. Int. J. Mol. Sci..

[18] Chen S.Y., Hwang J.S., Sung F.C., Lin C.Y., Hsieh C.J., Chen P.C., Su T.C. (2017). Mono-2-ethylhexyl phthalate associated with insulin resistance and lower testosterone levels in a young population. Environ. Pollut..

[19] Kang Y., Park J., Yoon K. (2019). Association between urinary phthalate metabolites and obesity in adult Korean population: Korean National Environmental Health Survey (KoNEHS), 2012–2014. Ann. Occup. Environ. Med..

[20] Huang Y.-C., Huang P.-R., Lo Y.-T.C., Sun C.-W., Pan W.-H., Wang S.-L., Huang H.-B. (2021). Food Processing and Phthalate Exposure: The Nutrition and Health Survey in Taiwan (1993–1996 and 2005–2008). Front. Nutr..

[21] American Diabetes Association (2026). Children and Adolescents: Standards of Care in Diabetes—2026. Diabetes Care.

[22] Weiner A., Zhang M., Ren S., Tchang B., Gandica R., Murillo J. (2023). Progression from prediabetes to type 2 diabetes mellitus in adolescents: A real world experience. Front. Clin. Diabetes Healthc..

[23] Nam D.J., Kim Y., Yang E.H., Lee H.C., Ryoo J.-H. (2020). Relationship between urinary phthalate metabolites and diabetes: Korean National Environmental Health Survey (KoNEHS) cycle 3 (2015–2017). Ann. Occup. Environ. Med..

[24] Mitra T., Sruthi A.H.A.K., Thangaraju R., Mani S.S.M., Kumari S.R., Janardhanan R. (2025). Impact of phthalate exposure on gestational diabetes mellitus: A systematic review. Front. Endocrinol..

[25] Liang Y., Li X. (2025). Individual and joint effects of exposure to phthalates and the risk of cardiovascular disease in the chronic kidney disease population: NHANES 2005–2018. Front. Public Health.

[26] Lind P.M., Zethelius B., Lind L. (2012). Circulating levels of phthalate metabolites are associated with prevalent diabetes in the elderly. Diabetes Care.

[27] Wang Z., Deng Y., Gao S., Lin Z., Zheng Z., Fang Q., Zhan M., Sun T., Huang G., Geng X. (2023). Association of urinary phthalate metabolites with all-cause and cardiovascular disease mortality among adults with diabetes mellitus: National Health and Nutrition Examination Survey 2005–2014. Front. Public Health.

[28] Peng M.Q., Karvonen-Gutierrez C.A., Herman W.H., Mukherjee B., Park S.K. (2023). Phthalates and incident diabetes in midlife women: The study of women’s health across the nation (SWAN). J. Clin. Endocrinol. Metab..

[29] Stahlhut R.W., van Wijngaarden E., Dye T.D., Cook S., Swan S.H. (2007). Concentrations of urinary phthalate metabolites are associated with increased waist circumference and insulin resistance in adult U.S. males. Environ. Health Perspect..

[30] Dong R., Chen J., Zheng J., Zhang M., Zhang H., Wu M., Li S., Chen B. (2018). The role of oxidative stress in cardiometabolic risk related to phthalate exposure in elderly diabetic patients from Shanghai. Environ Int..

[31] Kim J.H., Park H.Y., Bae S., Lim Y.H., Hong Y.C. (2013). Diethylhexyl phthalates is associated with insulin resistance via oxidative stress in the elderly: A panel study. PLoS ONE.

[32] Braun J.M. (2017). Early-life exposure to EDCs: Role in childhood obesity and neurodevelopment. Nat. Rev. Endocrinol..

[33] Franceschi C., Campisi J. (2014). Chronic inflammation (inflammaging) and its potential contribution to age-associated diseases. J. Gerontol. A Biol. Sci. Med. Sci..

[34] Guppy M., Thomas E.T., Glasziou P., Clark J., Jones M., O’Hara D.V., Doust J. (2024). Rate of decline in kidney function with age: A systematic review. BMJ Open.

[35] Corriere M., Rooparinesingh N., Kalyani R.R. (2013). Epidemiology of diabetes and diabetes complications in the elderly: An emerging public health burden. Curr. Diabetes Rep..

[36] Hackett R.A., Moore C., Steptoe A., Lassale C. (2018). Health behaviour changes after type 2 diabetes diagnosis: Findings from the English Longitudinal Study of Ageing. Sci. Rep..

[37] Grün F., Blumberg B. (2009). Endocrine disrupters as obesogens. Mol. Cell. Endocrinol..

[38] Kang J., Cho S.-Y., Yoon S. (2023). Relationship between the use of plastics in refrigerator food storage and urine phthalate metabolites: The Korean National Environmental Health Survey (KoNEHS) cycle 3. Ann. Occup. Environ. Med..

[39] Hernández-Díaz S., Mitchell A.A., Kelley K.E., Calafat A.M., Hauser R. (2008). Medications as a potential source of exposure to phthalates in the U.S. population. Environ. Health Perspect..

[40] Infurna F.J., Staben O.E., Lachman M.E., Gerstorf D. (2021). Historical change in midlife health, well-being, and despair: Cross-cultural and socioeconomic comparisons. Am. Psychol..

[41] Hill-Briggs F., Adler N.E., Berkowitz S.A., Chin M.H., Gary-Webb T.L., Navas-Acien A., Thornton P.L., Haire-Joshu D. (2020). Social determinants of health and diabetes: A scientific review. Diabetes Care.

[42] World Health Organization Social Determinants of Health. https://www.who.int/news-room/fact-sheets/detail/social-determinants-of-health.

[43] Dodson R.E., Udesky J.O., Colton M.D., McCauley M., Camann D.E., Yau A.Y., Adamkiewicz G., Rudel R.A. (2017). Chemical exposures in recently renovated low-income housing: Influence of building materials and occupant activities. Environ. Int..

[44] Buckley J.P., Kim H., Wong E., Rehkopf C.M. (2019). Ultra-processed food consumption and exposure to phthalates and bisphenols in the US National Health and Nutrition Examination Survey, 2013–2014. Environ. Int..

[45] Baker B.H., Melough M.M., Paquette A.G., Barrett E.S., Day D.B., Kannan K., Nguyen R.H.N., Bush N.R., LeWinn K.Z., Carroll K.N. (2024). Ultra-processed and fast food consumption, exposure to phthalates during pregnancy, and socioeconomic disparities in phthalate exposures. Environ. Int..

[46] Moermond C.T.A., Puhlmann N., Brown A.R., Owen S.F., Ryan J., Snape J., Venhuis B.J., Kümmerer K. (2022). GREENER pharmaceuticals for more sustainable healthcare. Environ. Sci. Technol. Lett..

